# Biologically synthesized metal nanoparticles: recent advancement and future perspectives in cancer theranostics

**DOI:** 10.4155/fsoa-2017-0035

**Published:** 2017-05-26

**Authors:** Sudip Mukherjee, Chitta Ranjan Patra

**Affiliations:** 1 Department of Chemical Biology, CSIR-Indian Institute of Chemical Technology, Uppal Road, Tarnaka, Hyderabad 500007, India; 2 Academy of Scientific & Innovative Research (AcSIR), Training & Development Complex, CSIR Campus, CSIR Road, Taramani, Chennai 600 113, India

**Keywords:** biosynthesis, cancer, green chemistry approach, metal nanoparticles, theranostics

Over the last two decades, nanotechnology has formed the most promising discipline within science likely to present solutions for the growing challenges in biomedical applications such as drug delivery, therapeutics and diagnostics [1–3]. Synthesis of metal nanoparticles, especially gold, silver and platinum (gold: AuNPs; copper, silver: AgNPs, platinum and quantum dots), with a particular shape, size and morphology is an important area of modern research. These nanoparticles are extensively used as an alternative strategy for cancer theranostics (therapeutics + diagnostics) due to their unusual physical, chemical, electronic and biological properties. Hence, economically cheap and eco-friendly methods for the synthesis of metal nanoparticles that could be useful for cancer theranostics are becoming an emerging field in biomedical research. In this context, biologically synthesized nanoparticles (b-NPs) using various biological sources (such as plants and microbes) overcome several disadvantages over chemically synthesized nanoparticles (c-NPs) [4–8]. Therefore, b-NPs are used for several biomedical applications especially in the area of cancer theranostics.

Several groups including our own demonstrated the cancer cell imaging ability of b-NPs using fluorescence and computed tomography imaging techniques [9,10]. These b-NPs demonstrated powerful imaging abilities and could be used for cancer diagnostics applications. Green synthesized metal nanoparticles or b-NPs further play an important role for the delivery of drugs or therapeutic agents, and show various advantages over conventional chemical-based drug delivery systems. Several phytochemicals including proteins, lipids, carbohydrates create an external biomatrix around the metal nanoparticle surface that helps in facile and effective binding of drug molecules, thus avoiding the use of any external chemical capping agents. Dhar *et al*. demonstrated drug delivery efficacy of gold nanoparticles (synthesized from gellan-gum and sophorolipid-gellan gum) conjugated US FDA doxorubicin in human glioma cell lines (LN-229 and LN-18) and a human glioma stem cell line (HNGC-2) [11,12]. Recently, our group demonstrated the *in vitro* and *in vivo* delivery efficacy of doxorubicin using b-AuNPs and b-AgNPs in various cancer cell lines and in a mouse melanoma tumor model [8,13]. Moreover, Ganeshkumar *et al*. demonstrated the targeted delivery of 5-fluorouracil using folic acid conjugated b-AuNPs (derived from using the fruit peel extract of *Punica granatum*) in MCF-7 breast cancer cell lines with high targeting efficacy [14]. Taken together, these recent published reports demonstrated the drug delivery efficacy of b-NPs in cancer therapy. Another successful biomedical application is the use of b-NPs as anticancer agents due to the presence of medicinally active anticancer phytochemicals (polyphenols, flavonoids, taxol, isoflavons, etc.) in the bioresources, which attach over the nanogold surface during the biosynthesis process. However, the chemically synthesized nanoconjugates barely show any therapeutic activity. Hence, b-NPs demonstrates a unique advantage over chemically modified nanoparticles (NPs). Recently, several groups including ours demonstrated the anticancer activities of b-NPs (b-AuNPs and b-AgNPs) derived from various bioresources in various cancer cell lines [7,9,15]. We demonstrated the anticancer activity of *in situ* one pot synthesized b-AuNPs and b-AgNPs using leaf extract of *Olax scandens* [9,16]. Importantly, b-AgNPs synthesized using the leaf extract of *Olax scandens* showed 4-in-1 biomedical applications – anticancer, antibacterial, biocompatible and imaging facilitator [9]. Our group also illustrated the anticancer activity of b-AuNPs derived from *Lantana montevidensis* leaf extract [7]. *Lantana montevidensis* contains various active anticancer phytochemicals (cirsilineol, eupatorine, apigenin, hispidulin, β-caryophyllene and eupafolin), which facilitate the potent anticancer activity. Very recently, Fazal *et al*. demonstrated excellent photothermal properties of anisotropic b-AuNPs (derived from *Theobroma cacao* [cocoa] seeds extract) in epidermoid carcinoma A431 cells using laser power density of 6 W/cm^2^ [10]. The b-AuNPs showed near-IR absorbance at 700–1000 nm that facilitated tremendous photothermal therapy against A431 cancer cells.

Recently, our group demonstrated that b-AuNPs synthesized from leaf extract of *Peltophorum pterocarpum*, the ‘yellow flame tree’, did not result in any significant changes in tissue histology (such as necrosis, inflammation, etc.) of various vital organs (liver, spleen, kidney, lung, heart, uterus, brain and thymus) or serum biochemical parameters (total glucose, cholesterol, SGPT, SGOT, triglycerides, urea nitrogen and uric acid) for nanoparticle-treated mice compared with an untreated control group after seven consecutive intraperitoneal injections at a dose of 10 mg/kg/b.w. of mouse [8]. However, notable toxicity symptoms were observed in the mouse group treated with chemically synthesized pegylated AuNPs under similar experimental conditions. This indicates that b-NPs showed higher biocompatibility and tolerability compared with c-NPs, which can be securely utilized for several biomedical applications including cancer theranostics, drug delivery and bioimaging. Altogether, numerous reports including the above-mentioned literature support the cancer theranostic applications of biocompatible b-NPs.

## Advantages & disadvantages of biosynthesis over chemical synthesis

### Advantages

Green chemistry based synthesis of metal nanoparticles has several unique advantages over the chemical-based synthesis approach. The mode of synthesis is easy (one-pot synthesis), fast, cost effective and eco-friendly. Moreover, the large and easy availability of the biological precursors (plants, microbes) helps in developing large scale-up technology. Various polyphenols and proteins present in the bioresources act as the reducing agent that minimizes the use of external chemical hazardous reducing agents and hence the toxicity. The green synthesis procedure does not require any additional capping agents or stabilizing agents, which further reduces the cost and simplifies the synthetic process. The b-NPs have ease of surface functionalization, which helps in decorating them with any imaging, therapeutic or other drug molecules. They are also generally found to be highly stable and monodispersed as compared with their chemical synthesized counterpart, which increases their utility toward various biomedical applications. Green synthesis procedures often do not need any special requirements and can be performed in room temperature conditions. More importantly, a large pool of pharmacologically active bioresources and potent phytochemicals conjugate with the b-NPs during the synthesis process, which make the b-NPs biologically active. This eliminates the requirements of additional attachments of drugs or any imaging agents externally that further reduces the complexity and cost and increases the therapeutic efficacy. Furthermore, the presence of diverse pharmacologically active biomolecules/phytochemicals in a single bioresource helps in the formation of multifunctional NPs that show diverse biomedical applications using the single entity.

### Disadvantages

Although the green chemistry approach has several advantages over chemical synthetic approaches still there are some major concerns that need to be resolved before successful commercial applications of these methodologies at a large industrial scale. The major problems include: the possible ecological imbalance in the natural bioresources (microbes, plants, etc.) due to excessive utilization; difference in the concentrations of active biomolecules/phytochemicals due to seasonal or climate changes, which affects the bioactivity or synthesis procedures; several NPs may not be prepared using biosynthesis approach due to the requirement of strong reducing agents; identification and isolation of bio-active molecules for cancer theranostics present in the plant leaf extract or other bioresources is a hurdle due to presence of a mixture of phytochemicals; attachment of desired or selective active molecules with b-NPs during synthesis is not possible most of the time; and synthesis of good manufacturing practice grade product needs a detailed investigation.

## Future perspective & challenges

Nanomedicine is the discipline of science that is likely to contribute significantly and imminently to overcoming the challenges and obstructions in the field of healthcare and medicine. There is a demand to develop use of green chemistry approaches compared with the conventional chemical nanofabrication techniques owing to the several advantages mentioned earlier. The green chemistry based approach to the synthesis of nanoparticles has proven to be eco-friendly, cost effective and multifunctional, with high scalability and stability. In the last two decades, the significant development of multifunctional b-NPs was demonstrated for biomedical applications, especially for cancer theranostics. The future of green synthesis is incredibly bright considering its low cost and high sustainability. However, there are several concerns including acute and chronic toxicity studies, immunogenicity, pharmacokinetics, pharmacodynamics, biodegradability, biodistribution, clearance, nanoparticles diffusion, uptake, scalable industrial production and disease-targeting ability that are yet to be addressed in detail before the clinical application of b-NPs [17].

Notably, the toxicity concerns for b-NPs have been observed to be low compared with c-NPs. Several groups including our own has reported the *in vitro* and *in vivo* biocompatibility of b-NPs [8–10,18]. Furthermore, b-NPs were found to be adsorbed less in serum proteins present in the blood plasma that indicates considerable *in vivo* stability [19]. However, there are no published reports presenting the degradability, clearance, immunogenicity, pharmacokinetics and long-term toxicity of b-NPs. Some recent reports demonstrated the metabolic degradability of chemically synthesized AuNPs and silica nanoparticles through liver and hepatocytes, which were further observed to be excreted by feces and urine [20]. Hence, detailed and careful studies need to be performed before understanding of the exact mechanistic pathways of clearance and biodegradability of b-NPs.

For the time being, it can be concluded that the future of b-NPs looks incredibly bright compared with c-NPs owing to its cheaper cost and eco-friendliness. The large pool of bioresources, if properly utilized (for the production of b-NPs along with their medicinal activities), could enable b-NPs to become a game changer in the coming future. However, identification of medicinally active phytochemicals is a time consuming, challenging and complex procedure that may halt the rapid progress of b-NPs. Hence, it will take some time (5–10 years) to develop a rational and efficient method for the identification and isolation of the potential active phytochemicals (present in the large mixture of bioresources) that can be utilized for the synthesis of b-NPs. Nevertheless, the toxicity, high cost and degradability concerns associated with c-NPs make it hard for their successful clinical applications. Although the mechanistic pathways underlying b-NP diffusion, uptake, excretion and activity remain uncertain, the impending biological response shown by b-NPs exhibits substantial cancer theranostic applications that make them a potential contender to widely replace the complicated and costly c-NPs in the coming years. Hence, careful *in vitro* and *in vivo* characterization followed by preclinical and clinical safety and therapeutic analysis could establish the b-NPs as proficient and alternative candidates toward cancer theranostics applications in the near future.
